# The adaption of the Chinese version of the COVID Stress Scales as a screening instrument of stress: Psychometric properties during the COVID-19 pandemic

**DOI:** 10.3389/fpubh.2022.962304

**Published:** 2022-08-17

**Authors:** Lu Xia, Qiaoping Lian, Haibo Yang, Daxing Wu

**Affiliations:** ^1^Medical Psychological Center, The Second Xiangya Hospital, Central South University, Changsha, China; ^2^Tianjin Normal University, Academy of Psychology and Behavior, Tianjin, China; ^3^Medical Psychological Institute of Central South University, Changsha, China; ^4^National Clinical Research Center on Mental Disorders, Changsha, China

**Keywords:** COVID Stress Scales, adaptation, validation, stress of disease, COVID-19 pandemic

## Abstract

The COVID Stress Scales (CSS) was used to access related distress concerning Corona Virus Disease 2019 (COVID-19). Based on China's epidemic prevention and control policies during the COVID-19 pandemic, the adaption of the Chinese version of the CSS was developed. Our study evaluated the reliability and validity of the Chinese adapted version of the CSS during the COVID-19 pandemic. An online survey was employed to construct a national sample of 2,116 participants in Chinese mainland. We examined the factor structure, internal consistency, convergent validity, discriminant validity, and concurrent validity. The results demonstrated that the six-factor solution for the Chinese adaptation of the CSS proved a good fit with the data after comparing the factor structure with the five-factor model. The six-factor model had good reliability and supported good convergent, discriminant, and concurrent validity of the CSS Chinese adaption. Overall, our findings supported the Chinese adapted version of the CSS as a psychometrically sound measure of stress during the COVID-19 pandemic in China.

## Introduction

The coronavirus disease 2019 (COVID-19) outbreak became a worldwide health emergency. In December 2019, the first cases of pneumonia of unknown origin were identified in Wuhan, China. The WHO confirmed a new coronavirus was the cause of pneumonia in Wuhan (1). The complexity and uncertainty of the pandemic threatened human physical health and mental health (2). Plenty of studies reported negative psychological effects such as posttraumatic stress symptoms, fear, and confusion (3). The stress caused by the virus increased during a lock-down period at home (4, 5). Besides, the resurgence of COVID-19 could exacerbate the psychological impacts of the pandemic (6). Due to the social and economic uncertainty associated with the COVID-19 pandemic, certain groups such as young people and women had a risk of suicide during the height of the pandemic (7). Another study reported that people might experience mild to severe depression and anxiety symptoms while the outbreak continued (8). Their positive emotion progressively decreased over time (9).

In response to the unprecedented psychological impact of the current pandemic, Taylor suggested that developing a measure of COVID-19-related stress and anxiety was an urgent need during the pandemic (10). After examining the relevant literature and consulting experts, they developed the COVID Stress Scales (CSS) to measure COVID-19-related stress (10). The CSS contained six domains: (1) the worries about the dangerousness of COVID-19, (2) fears about COVID socio-economic consequences (e.g., fears of disruption in the supply chain), (3) COVID-19-xenophobia (e.g., fears that foreigners are sources of COVID-19), (4) the worries about being infected by COVID-19-related contamination (i.e., objects, surfaces), (5) traumatic stress symptoms (e.g., nightmares relating to COVID-19), and (6) COVID-19-related checking (e.g., checking news media). The initial psychometric evaluation of the CSS demonstrated good reliability, convergent and discriminant validity (10). The CSS total and subscale scores had shown high internal consistency and test-retest reliability, and the internal consistency of the total score had been reported as 0.95 (10). There were controversial about the factor structure. The initial development was constructed by examining relevant literature and consulting experts, which contained six domains. However, the results from the exploratory factor analysis (EFA) indicated a five-factor solution, COVID-related danger scale and contamination scale loaded on the same factor (10). The five-factor solution was also confirmed in other studies (11, 12). Although Taylor proposed a five-factor model of CSS that was based on a large collection of research evidence, plenty of studies applying confirmatory factor analysis (CFA) had shown a good fit for the six-factor model with the studied population (13–16). Besides, we found that the CSS-Arabic version supported the five-factor solution in the Egyptian and Saudi contexts (11). While it also supported a six-factor solution in a Palestinian context after excluding five items (14). Nevertheless, the CSS Spanish translation proved that the six-factor model was better than the five-factor model and the six-factor model fitted the data well (16). Above all the current studies which were proved to be valid and reliable were conducted in other cultural contexts, which hadn't been adapted and validated in the Chinese population during the COVID-19 pandemic. Standards for Educational and Psychological Testing recommended that evidence of validity should be clarified for each intended use of the test score among the targeted population and for specific procedures (17). It was necessary to confirm the factor structure in the Chinese context.

To reduce the rapid spread of COVID-19 across the world, China decided to suspend the entry into China by foreign nationals holding visas or residence permits temporarily on 26 March 2020 (18). Then a portion of foreign nationals was allowed to enter China after 23 September 2020 according to the announcement, but other measures in the Announcement issued on March 26 continued to be implemented (19). Regarding the epidemic prevention and control policies in China, the foreigners in the CSS xenophobia domain were defined as COVID-19-related personnel, including personnel living in high-risk areas or passing through them, the frontline medical staff, epidemic prevention workers, and other relevant personnel.

Briefly, the main goal of our study was to adapt the CSS to Chinese and to determine the psychometric properties of the Chinese population during the COVID-19 epidemic. Firstly, we explored the factor structure and compared the different factor structures of the adaptation of the Chinese version of the CSS. Additionally, internal consistency values were calculated for the reliability of the Chinese adapted version of the CSS. Psychometric studies of the Persian and Arabic versions of the CSS were based on the Classical Theory of Tests (CTT), which emphasized the evaluation of internal consistency and construct validity of an instrument in a general way (20). In our study, we used the CTT to evaluate the internal consistency and construct validity of the CSS Chinese adaptation (21). Average Variance Extracted (AVE), Composite Reliability (CR), and the square root of the AVE were used to assess convergent validity and discriminant validity. Finally, we calculated the correlations between the CSS and other scales including the Coronavirus Anxiety Scale (CAS), the Fear of COVID-19 Scale (FCV-19S), and depression anxiety stress scales (DASS-21) to examine its concurrent validity.

## Methods

### Participants and procedure

Over 1 week period in August 2021, a cross-sectional online survey was conducted in Chinese mainland through the WeChat public platform following the electronic research methodology guideline (22) to prevent the spread of COVID-19 through contact. All participants using WeChat might see this survey, and answer the questionnaire by scanning the two-dimensional barcodes of the questionnaire address or clicking the relevant link. Electronic informed consent was obtained from each participant before starting the investigation. All participants were provided with anonymity and confidentiality of their data, and they were informed about the nature, purpose, and procedure of the study. Participants could withdraw from the survey at any moment without providing any justification. This web-based questionnaire was completely voluntary and non-commercial. Participants agreed to the online informed consent statement and completed the questionnaires. After completing the scales, every participant would receive a reward, which contained an individual report and reward (1-3 CNY randomly). The research was approved by the ethics committee of the Second Xiangya Hospital of Central South University.

To ensure the data quality, questionnaires were valid if they met the following criteria. The inclusion criteria included: (1) time for each item completion more than 2 seconds; (2) the participants aged 18 and above, and (3) the item responses were not consecutive identical. Finally, 2,116 questionnaires were included in the final analysis.

### Measures

#### The COVID Stress Scales (CSS)

The CSS was a 36-item self-report instrument designed to assess COVID-19-related stress and anxiety symptoms over the past week. Items were scored on a 5-point Likert scale from 0 (not at all) to 4 (extremely). Higher scores indicated a higher level of stress. The transcultural and lingual adaption from English to Chinese was performed using Beaton and colleagues' methods (23). The translation process was composed of several steps based on the Brislin translation model (24).

We used a forward-backward translation. The translation procedures were as follows. Firstly, the CSS was translated from English into Chinese by two native Chinese-speaking researchers with high English proficiency from the research team independently. Subsequently, two psychology experts reviewed the translated version concerning its content accuracy, semantic equivalence, and sentence structure. Modifications were made after the group reached a consensus. Some minor revisions were made during the translation process. Lastly, the Chinese-adapted version of the CSS was back-translated into the English language again. The back-translated and original versions were compared to ensure accuracy. Our research team reviewed and checked the translation. Finally, the adaption of the Chinese version of the CSS was confirmed.

Considering epidemic prevention and control policies in China, the foreigners in the CSS xenophobia domain were changed to COVID-19-related personnel which included personnel living in or passing through high-risk areas, the frontline medical staff, epidemic prevention workers, and other relevant personnel. Six items of the xenophobia domain were adapted. The amended items were (a) “ *I am worried that COVID-19-related personnel is spreading the virus*”, (b) “*If I met COVID-19-related personnel, I'd be worried that they might have the virus*”, (c) “*I am worried about contacting COVID-19-related personnel because they might carry the virus*”, (d) “*If the COVID-19-related personnel doesn't take care of their hygiene, I'm worried they'll spread the virus*”, (e) “*If I went to a restaurant where COVID-19-related personnel has been, I'd be worried about catching the virus*”, and (f) “*If I was in an elevator with COVID-19-related personnel, I'd be worried that they're infected with the virus*“.

#### The Coronavirus Anxiety Scale (CAS)

The CAS was a 5-item self-report instrument designed to assess the levels of COVID-19 anxiety (25). The CAS had good reliability and validity (2, 26, 27). It was a brief mental health screener to measure current anxiety over the last 2 weeks. Items were rated on a 5-point Likert scale ranging from 0 (not at all) to 4 (nearly every day over the last 2 weeks). In our study, Cronbach's alpha internal consistency coefficient was 0.95.

#### The Fear of COVID-19 Scale (FCV-19S)

The FCV-19S was a 7-item instrument to measure an individual's fear of COVID-19 (28). Items were scored on a 5-point Likert scale from 1 (strongly disagree) to 4 (strongly agree). The higher score indicated a higher level of fear. The Chinese version of the FCV-19S had good internal consistency reliability (Cronbach's alpha = 0.92) and validity (29). In our study, Cronbach's alpha internal consistency coefficient was 0.95.

#### Depression Anxiety Stress Scales (DASS-21)

DASS-21 was a 21-item instrument to measure the experiences of depression, anxiety, and stress over the past week (30). It consisted of three subscales: depression, anxiety, and stress. Each subscale included seven items. Items were rated on a 4-point Likert scale ranging from 0 (not at all) to 3 (strongly agree). The scale had been validated in China (31). In our study, Cronbach's alpha internal consistency coefficient was 0.98.

### Statistical analysis

Data were analyzed with the SPSS 26.0 and Amos 26.0. We assessed internal consistency reliability using Cronbach's alpha(α), McDonald's omega(ω), and the Spearman-Brown formula(accepted value ≥0.70) using the CTT (21). Values equal to or greater than α = 0.70 ω = 0.70 were considered satisfactory (32, 33). CFA was to test the hypothesized factor structures obtained from the Canadian and American samples (10) and the Arabic sample (14) separately. Model fit was assessed using comparative fit index (CFI), Tucker-Lewis index (TLI), normed fit index (NFI), incremental fit index (IFI), root-mean-square-error of approximation (RMSEA), and standardized root-mean residual (SRMR). The CFI, NFI, IFI, and TLI values ≥0.90 suggested the good fit model (34). Additionally, the RMSEA value between 0.06 and 0.08 and the SRMR value ≤ 0.08 suggested a better-fitted model (35). The smallest Akaike information criterion (AIC), Bayesian information criterion (BIC), and expected cross-validation Index (ECVI) indicated the model with the best fit (36).

Besides, an instrument's convergent validity could be determined by examining two variables, the AVE of the latent variable and the measure's CR. Convergent validity could be considered adequate when the AVE value was ≥0.50, the CR value was ≥0.70 (37, 38). The discriminant validity was measured by the square root of the AVE. The square root of the AVE in each factor was better than the correlation coefficient value of the factor with other factors indicating it had good discriminant validity (39).

Finally, criterion validity was made up of two subcategories: predictive and concurrent. Concurrent validity was demonstrated when two assessments agreed or a new measure was compared with one already considered valid (40). Previous studies had revealed that pandemic-related anxiety was distinct from anxiety-related traits (41, 42), but few studies measured pandemic-related anxiety as concurrent validity. Therefore, concurrent validity was a necessary content in criterion validity. Thus, concurrent validity was measured by calculating the Pearson product-moment correlation coefficient between the CSS and other correlated scales (CAS, FCV-19S, DASS-21).

## Results

### Demographic variable

We summarized the participants' characteristics in Table 1. A total of 2,116 participants were included in the study. Respondents aged from 18 to 68 (M = 31.21, SD = 9.51) and the females accounted for 59.0% of the total sample. Most of the participants got vaccinated (67.6%). Among the sample, the monthly income of the majority of subjects was 5,000–9,999 CNY (42.9%).

**Table 1:** Sample characteristics (*n* = 2,116).

**Characteristic**	**Variable**	**M**	**SD**
Age		31.21	9.51
		*Count*	*Percent*
Gender	Male	868	41.0
	Female	1,248	59.0
Education level	Junior school and below	288	13.6
	Senior school	738	34.9
	Bachelor	982	46.4
	Master and above	108	5.1
Marital status	Single	866	40.9
	Married	1,204	56.9
	Divorced	37	1.8
	Widowed	9	0.4
Monthly income level (CNY)	2,000 or less	398	18.8
	2,000–4,999	623	29.4
	5,000–9,999	908	42.9
	10,000 or more	187	8.9
Occupation	Healthcare workers^a^	507	24.0
	Enterprise or institution workers^b^	600	28.3
	Teachers or students^c^	514	24.3
	Others^d^	495	23.4
Vaccination	Not Vaccinated	686	32.4
	Vaccinated	1,430	67.6

a *Included doctors, nurses, disease control staff, medical departmental managers, and psychological counselors*.

b *Included government personnel, community staff, volunteers, social workers, and policies*.

c *Included teachers or students from universities, middle schools, or elementary schools*.

d *Included freelancers, retirees, and other relevant staff*.

*M, mean; SD, standard deviation*.

### Construct validity

**Figure 1:**
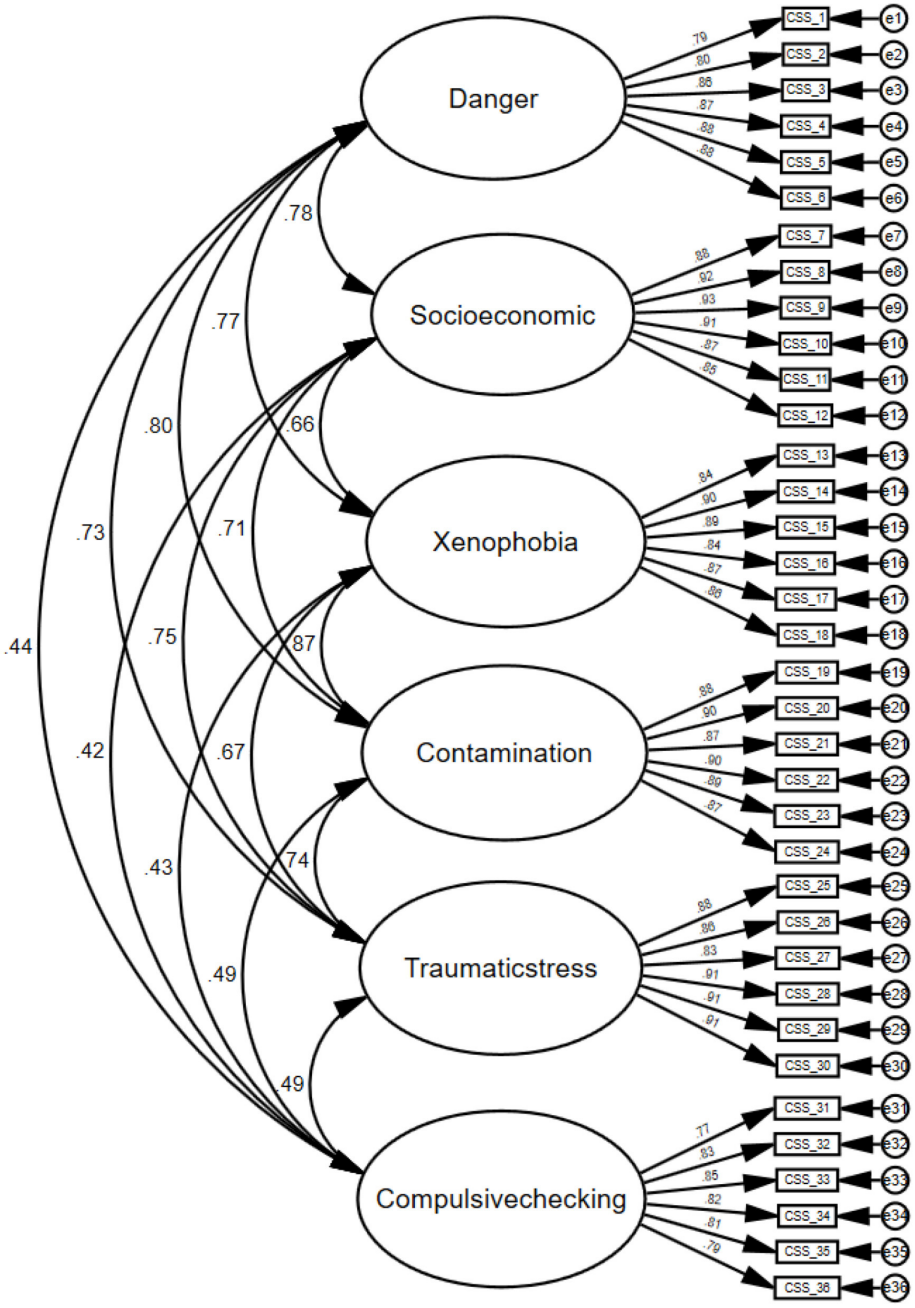
Confirmatory factor analysis of the COVID Stress Scales.

We tested the models mentioned in the literature to verify the structural validity of the CSS Chinese adapted version. One was the six-factor model, which included danger (D), socio-economic consequences (SE), xenophobia (X), contamination (C), traumatic stress (T), and compulsive checking (CH). The other was the five-factor model, which combined danger (D) and contamination (C) into one factor. When modeling ordered categorical data, the research seemed to indicate that if there were a large number of ordered categories the data could be treated as continuous. Finney and DiStefano recommended treating the data as continuous and employing maximum likelihood estimation if the variables had five categories or more, the data were approximately normally distributed (43). Thus, we used the maximum likelihood to estimate each model in our study. The CFA was conducted to determine the goodness-of-fit of the six-factor model with 36 items (Figure 1).

As was shown in Table 2, all resulting incremental indices in the six-factor model were greater than those in the five-factor model and exceeded the 0.90 level, indicating a better model fit in the six-factor model. The RMSEA value was 0.067 (90 % confidence interval:0.065–0.068) in the six-factor model; in the five-factor model, the RMSEA value was 0.085 (90 % confidence interval:0.083–0.086). The SRMR value in the six-factor model was 0.038, while in the five-factor solution was 0.047. The AIC, BIC and ECVI in the six-factor model [AIC = 6,213.648, BIC = 6,705.832, and ECVI = 2.938 (90 % confidence interval: 2.821–3.057)] smaller than those [AIC = 9,610.543, BIC = 10,074.440, and ECVI = 4.544 (90 % confidence interval:4.397–4.694)] in five-factor model indicated the six-factor model with the best fit.

**Table 2:** Fit indices of various structural models for confirmatory factor analysis (*n* = 2,116).

**Model**	**χ2**	***df***	**RMESA [90%CI]**	**CFI**	**TLI**	**NFI**	**IFI**	**SRMR**	**AIC**	**BIC**	**ECVI [90%CI]**
Six-factor model	6,039.648	579	0.067 [0.065-0.068]	0.935	0.929	0.929	0.935	0.038	6,213.648	6,705.832	2.938 [2.821-3.057]
Five-factor model	9,446.543	584	0.085 [0.083-0.086]	0.895	0.886	0.889	0.895	0.047	9,610.543	10,074.440	4.544 [4.397-4.694]

*df, degrees of freedom; RMSEA, root-mean-square-error of approximation; CI, confidence interval; CFI, comparative fit index; TLI, Tucker-Lewis index; NFI, normed fit index; IFI, incremental fit index; SRMR, standardized root-mean residual; AIC, Akaike information criterion; BIC, Bayesian information criterion; ECVI, expected cross-validation Index*.

### Internal consistency reliability

Cronbach's alpha (α), McDonald's omega (ω), and Spearman-Brown coefficient were calculated for the internal consistency of the CSS Chinese adaptation. Cronbach's alpha was 0.97, McDonald's omega was 0.97, and split-half reliability through the Spearman-Brown formula was 0.90, indicating the high reliability of the CSS Chinese adapted version. The Cronbach's coefficients of subscale scores and McDonald's Omega subscale value in terms of the six-factor model were summarized in Table 3. The dimensions of Danger (α = 0.94; ω = 0.94), Socioeconomic consequences (α = 0.96; ω = 0.96), Xenophobia (α = 0.95; ω = 0.95), Contamination (α = 0.96; ω = 0.96), Traumatic stress (α = 0.95; ω = 0.96), and Compulsive checking (α = 0 .92; ω = 0.92) had adequate reliability indices.

**Table 3:** Normative data and reliability indices for the COVID Stress Scales and its subscales (*n* = 2,116).

**Scales**	**M**	**SD**	**Alpha**	**Omega**
COVID Stress Scales	29.58	26.71	0.97	0.97
COVID danger	4.56	5.38	0.94	0.94
COVID socio-economic consequences	3.12	5.25	0.96	0.96
COVID xenophobia	5.63	5.74	0.95	0.95
COVID contamination	4.88	5.45	0.96	0.96
COVID traumatic stress	2.73	4.57	0.95	0.96
COVID compulsive checking	8.67	6.04	0.92	0.92

*Alpha, Cronbach's alpha coefficient; Omega, McDonald's omega coefficient. M, mean; SD, standard deviation*.

### Convergent validity and discriminant validity

As shown in Table 4, the factor loading of each item on all corresponding subscales was better than 0.70 in the six-factor model. AVE was better than 0.50, and CR was better than 0.80. These results supported a good convergent validity of the Chinese adapted version of the CSS.

**Table 4:** Convergent validity (*n* = 2,116).

**Items**		**Factor**	**Estimate**	**CR**	**AVE**
CSS_1	< –	D	0.787	0.939	0.719
CSS_2	< –	D	0.802		
CSS_3	< –	D	0.855		
CSS_4	< –	D	0.875		
CSS_5	< –	D	0.884		
CSS_6	< –	D	0.880		
CSS_7	< –	SE	0.879	0.960	0.799
CSS_8	< –	SE	0.917		
CSS_9	< –	SE	0.927		
CSS_10	< –	SE	0.915		
CSS_11	< –	SE	0.871		
CSS_12	< –	SE	0.852		
CSS_13	< –	X	0.836	0.948	0.752
CSS_14	< –	X	0.895		
CSS_15	< –	X	0.894		
CSS_16	< –	X	0.844		
CSS_17	< –	X	0.872		
CSS_18	< –	X	0.859		
CSS_19	< –	C	0.876	0.955	0.781
CSS_20	< –	C	0.899		
CSS_21	< –	C	0.870		
CSS_22	< –	C	0.897		
CSS_23	< –	C	0.887		
CSS_24	< –	C	0.873		
CSS_25	< –	T	0.876	0.956	0.783
CSS_26	< –	T	0.861		
CSS_27	< –	T	0.831		
CSS_28	< –	T	0.911		
CSS_29	< –	T	0.915		
CSS_30	< –	T	0.911		
CSS_31	< –	CH	0.773	0.921	0.660
CSS_32	< –	CH	0.827		
CSS_33	< –	CH	0.848		
CSS_34	< –	CH	0.822		
CSS_35	< –	CH	0.814		
CSS_36	< –	CH	0.789		

*AVE, Average Variance Extracted; CR, Composite Reliability; CSS, COVID Stress Scales; D, danger; SE, socio-economic consequences; X, xenophobia; C, contamination; T, traumatic stress; CH, compulsive checking*.

As shown in Table 5, the square root of the AVE in each factor (D, SE, X, C, T, CH) was better than the correlation coefficient value of the factor with the other factor. According to Zait and Bertea, the results showed that it had a good discriminant validity (39).

**Table 5:** Discriminant validity (*n* = 2116).

	**D**	**SE**	**X**	**C**	**T**	**CH**
D	(0.848)					
SE	0.749^**^	(0.894)				
X	0.726^**^	0.638^**^	(0.867)			
C	0.766^**^	0.688^**^	0.837^**^	(0.884)		
T	0.704^**^	0.721^**^	0.637^**^	0.710^**^	(0.885)	
CH	0.417^**^	0.396^**^	0.408^**^	0.460^**^	0.461^**^	(0.813)

** * p < 0.01*.

*The numbers on the diagonal were the square root of the average variance extraction (AVE)*.

*D, danger; SE, socio-economic consequences; X, xenophobia; C, contamination; T, traumatic stress; CH, compulsive checking*.

### Concurrent validity

As shown in Table 6, there were strong positive correlations between the FCV-19S, the CAS, the DASS-21, and the CSS. The CSS total score was positively correlated with the FCV-19S (*r* = 0.73), the CAS (*r* = 0.66), the DASS-21 (*r* = 0.63), the DASS_D (*r* = 0.58), the DASS_A (*r* = 0.63) and the DASS_S (*r* = 0.62). The results provided an evidence of criterion validity.

**Table 6:** Pearson's correlations between the FCV-19S, the CAS, the DASS-21, and the CSS.

	**1**	**2**	**3**	**4**	**5**	**6**	**7**
1.DASS-21	1						
2.DASS_D	0.97^**^	1					
3.DASS_A	0.97^**^	0.91^**^	1				
4.DASS_S	0.97^**^	0.92^**^	0.92^**^	1			
5.FCV-19S	0.58^**^	0.53^**^	0.58^**^	0.57^**^	1		
6.CAS	0.55^**^	0.51^**^	0.56^**^	0.53^**^	0.52^**^	1	
7.CSS	0.63^**^	0.58^**^	0.63^**^	0.62^**^	0.73^**^	0.66^**^	1

** *Correlation is significant at the 0.01 level (2-tailed)*.

*DASS-21, depression anxiety stress scales; DASS_D, DASS-21 depression subscale; DASS_A, DASS-21 anxiety subscale; DASS_S, DASS-21 stress subscale; FCV-19S, The Fear of COVID-19 Scale; CAS, Coronavirus Anxiety Scale; CSS, COVID Stress Scales*.

## Discussion

During the COVID-19 pandemic, the scales which assessed general mental health might be underestimated or overestimated because they didn't assess specific symptoms associated with COVID-19 (44). In response to the current pandemic, Taylor suggested that developing a measure of COVID-19-related stress and anxiety was an urgent need during the pandemic (10). The current study aimed to examine the psychometric properties of the adaption of the Chinese version of the CSS. The study showed that the Chinese adapted version of the CSS had good internal consistency, convergent validity, discriminant validity, and concurrent validity. Besides, we tested the factor structure and found the Chinese adapted version fit the six-factor model. Overall, the results indicated that the Chinese adapted version of the CSS showed good psychometric properties.

In our study, the six-factor structure was supported by CFA in the Chinese adapted versions of the CSS. The findings echoed the previous researchers (14, 16) and the original authors' six scales construction (10) though inconsistent with the five-factor model reported by the original research (10) and other researchers (12, 45). The six-factor model presented adequate adjustment indices in our sample of participants (RMSEA = 0.067, SRMR = 0.038; CFI = 0.935; and TLI = 0.929). In contrast, five-factor model had worse adjustment indices (RMSEA = 0.085; SRMR = 0.047; CFI = 0.895; and TLI = 0.886). Besides CFI, TLI, RMSEA, and SRMR, we also compared other resulting incremental indices such as AIC, BIC, and ECVI in the study. The six-factor model (AIC = 6,213.648, BIC = 6,705.832; ECVI = 2.938) were smaller than those in the five-factor model (AIC = 9,610.543; BIC = 10,074.440; ECVI = 4.544), indicating the better model fit in the six-factor model. In our study, factor loadings ranged from 0.7–0.93, which was a higher range than reported. It was worth noting that factor loadings were even high, above what was recommended (46). The difference in the factor structure across different surveys might result from the difference in the population. The original validation study, utilizing parallel analyses, demonstrated that the five-factor solution was sufficiently stable for the Canadian and US community-based samples (10). This solution was replicated in Iran among persons with anxiety and obsessive-compulsive disorders (12). While our study was conducted in the Chinese population, similar to the results in the Peruvian context (16). The most confusing was that the CSS Arabic version supported the five-factor solution in the Egyptian and Saudi contexts (11). Meanwhile, it also supported the six-factor solution in a Palestinian context after excluding five items (14). We deduced that the danger and contamination factors were the separated factors in different populations. Danger and contamination subscales were merged and observed as a single construct in the five-factor model. Differences in the factor structure of the CSS might reflect a specific context or purpose of the CSS. Our findings suggested that in the Chinese population, a distinction between perceptions of the pandemic as dangerous and disrupting everyday functioning on the one hand, and getting exposure to virus in the immediate environment on the other hand, should be made. Besides, it was related to what Taylor suggested that people use various psychological factors when facing the threat of a pandemic, presenting adaptive behaviors, emotions, and defensive reactions linked to their psychological vulnerability (47).

The Chinese adapted version of the CSS and its subscales showed good internal consistency. The internal consistency was also been reported using the McDonald's omega coefficient, which was a more appropriate estimation measure that was based on factor loadings and was not influenced by sample size or the number of items on the scale (48). The internal consistency of the total (α = 0.97, ω = 0.97, Spearman-Brown coefficient = 0.90) and subscale scores (α = 0.92–0.96; ω = 0.92–0.96) were similar to those reported in previous psychometric studies of the CSS (11).

The convergent validity and discriminant validity of the Chinese adaption of the CSS were established in the study. The significant correlations between the subscales in the present study were close to the association reported by the original authors (10). In Taylor and his colleagues' (10) study, the convergent validity was evaluated through the correlations of the scales of the CSS with the pre-COVID trait measures such as anxiety and obsessive-compulsive (OC) symptoms. The discriminant validity was evaluated by the correlations of the general and pre-COVID traits. In our study, the convergent validity was assessed through AVE and CR, the factor loading of each item on all corresponding subscales was better than 0.70. AVE was better than 0.50, and CR was better than 0.80. The square root of the AVE in each factor was to access the discriminant validity. The results demonstrated that the square root of the AVE in each factor was greater than the correlation coefficient value of the factor with other factors. The results supported good convergent validity of the Chinese adapted version of the CSS through diverse statistical methods.

According to previous studies (49, 50), mental health was associated with stress measures. In our study, all correlations were significant between the FCV-19S, the CAS, the DASS-21, and the CSS. The findings supported the concurrent validity of the CSS. The correlation coefficients were significant as expected (*r* = 0.58–0.73). The correlations between the FCV-19S, the CAS, and the CSS were in line with other studies examining COVID-19-related scales (12, 41). Similar results might indicate that COVID-19-related health anxiety was distinct from anxiety traits (41, 42). The correlation with DASS-21 was in line with the previous study (14). The results suggested that more stress perceived by individuals under the COVID-19 pandemic might be associated with higher depression and anxiety symptoms (51).

Our study also had several limitations. Firstly, we conducted the online survey with self-report measurements due to the COVID-19 epidemic. Secondly, this study did not investigate other aspects of reliability and validity such as test-retest reliability and predictive validity. Evidence of test-retest reliability would be a benefit for longitudinal research in the future. Thirdly, future researchers could develop more objective stress measures, such as physiological stress indicators or known-groups validity (10) to investigate the criterion validity of the CSS.

## Conclusion

Regardless of all the above limitations, this study provided data supporting the psychometric properties of the adaption of the Chinese version of the CSS. The results supported the six-factor structure proposed in prior research. In conclusion, the Chinese adapted version of the CSS was a reliable and valid tool for assessing the stress of COVID-19 in China. The spread of coronavirus in China could amplify the risk of maladaptive and stressful symptoms in an already compromised living environment. Understanding stress-related and COVID-19-associated outcomes might be an urgent priority in China.

## Data availability statement

The raw data supporting the conclusions of this article will be made available by the authors, without undue reservation.

## Ethics statement

All procedures performed in this study involving human participants were following the ethical standards of the Committee of the Second Xiangya Hospital of Central South University, the American Psychological Association (APA, 2010), and the 2013 Helsinki Declaration. The patients/participants provided their written informed consent to participate in this study.

## Author contributions

DW conceived and designed the study. LX and QL performed the analysis. LX, QL, HY, and DW prepared and modified the manuscript. All authors were involved in the study conduction and contributed substantially to its revision and approved the submitted version.

## Funding

This study was supported by the 225 High-level Health Talents Training Project of Hunan Province of China and the Natural Science Foundation of Hunan Province (Grant No: 2016JJ4101).

## Conflict of interest

The authors declare that the research was conducted in the absence of any commercial or financial relationships that could be construed as a potential conflict of interest.

## Publisher's note

All claims expressed in this article are solely those of the authors and do not necessarily represent those of their affiliated organizations, or those of the publisher, the editors and the reviewers. Any product that may be evaluated in this article, or claim that may be made by its manufacturer, is not guaranteed or endorsed by the publisher.
