# Supplementary material for: The adaption of the Chinese version of the COVID Stress Scales as a screening instrument of stress: Psychometric properties during the COVID-19 pandemic
Source: Front Public Health. 2022 Aug 17;10:962304. doi: 10.3389/fpubh.2022.962304 (PMC9429793; doi:10.3389/fpubh.2022.962304)
Supplement: Supplementary file 1 [file Data_Sheet_1.docx]

**Appendix A**

COVID Stress Scales

*Appendix A.1. English Version*

**TableA1** Items of the COVID Stress Scales (English version)

| **The following asks about various kinds of worries that you might have experienced over the past seven days. In the following statements, we refer to COVID-19 as "the virus".** | Not at all | Slightly | Moderately | Very | Extremely |
| --- | --- | --- | --- | --- | --- |
| 1. I am worried about catching the virus. | 口 | 口 | 口 | 口 | 口 |
| 1. I am worried that basic hygiene (e.g., handwashing) is not enough to keep me safe from the virus. | 口 | 口 | 口 | 口 | 口 |
| 1. I am worried that our healthcare system is unable to keep me safe from the virus. | 口 | 口 | 口 | 口 | 口 |
| 1. I am worried that I can’t keep my family safe from the virus. | 口 | 口 | 口 | 口 | 口 |
| 1. I am worried that our healthcare system won’t be able to protect my loved ones. | 口 | 口 | 口 | 口 | 口 |
| 1. I am worried that social distancing is not enough to keep me safe from the virus. | 口 | 口 | 口 | 口 | 口 |
| 1. I am worried about grocery stores running out of food. | 口 | 口 | 口 | 口 | 口 |
| 1. I am worried about grocery stores running out of cold or flu remedies. | 口 | 口 | 口 | 口 | 口 |
| 1. I am worried about pharmacies running out of prescription medicines. | 口 | 口 | 口 | 口 | 口 |
| 1. I am worried about grocery stores running out of water. | 口 | 口 | 口 | 口 | 口 |
| 1. I am worried about grocery stores running out of cleaning or disinfectant supplies. | 口 | 口 | 口 | 口 | 口 |
| 1. I am worried that grocery stores will close down. | 口 | 口 | 口 | 口 | 口 |
| **The following asks about various kinds of worries that you might have experienced over the past seven days. In the following statements, we refer to COVID-19 as "the virus".** | Not at all | Slightly | Moderately | Very | Extremely |
| 1. I am worried that foreigners are spreading the virus in my country. | 口 | 口 | 口 | 口 | 口 |
| 1. If I met a person from a foreign country, I’d be worried that they might have the virus. | 口 | 口 | 口 | 口 | 口 |
| 1. I am worried about coming into contact with foreigners because they might have the virus. | 口 | 口 | 口 | 口 | 口 |
| 1. I am worried that foreigners are spreading the virus because they’re not as clean as we are. | 口 | 口 | 口 | 口 | 口 |
| 1. If I went to a restaurant that specialized in foreign foods, I’d be worried about catching the virus. | 口 | 口 | 口 | 口 | 口 |
| 1. If I was in an elevator with a group of foreigners, I’d be worried that they’re infected with the virus. | 口 | 口 | 口 | 口 | 口 |
| 1. I am worried that people around me will infect me with the virus. | 口 | 口 | 口 | 口 | 口 |
| 1. I am worried that if I touched something in a public space (e.g., handrail, door handle), I would catch the virus. | 口 | 口 | 口 | 口 | 口 |
| 1. I am worried that if someone coughed or sneezed near me, I would catch the virus. | 口 | 口 | 口 | 口 | 口 |
| 1. I am worried that I might catch the virus from handling money or using a debit machine. | 口 | 口 | 口 | 口 | 口 |
| 1. I am worried about taking change in cash transactions. | 口 | 口 | 口 | 口 | 口 |
| 1. I am worried that my mail has been contaminated by mail handlers. | 口 | 口 | 口 | 口 | 口 |
| **In the following statements, we refer to COVID-19 as "the virus". Please read each statement and indicate how frequently each problem has been for you during the past seven days.** | Never | Rarely | Sometimes | Often | Almost always |
| 25. I had trouble sleeping because I worried about the virus. | 口 | 口 | 口 | 口 | 口 |
| 26. I had bad dreams about the virus. | 口 | 口 | 口 | 口 | 口 |
| 27. I thought about the virus when I didn’t mean to. | 口 | 口 | 口 | 口 | 口 |
| 28. Disturbing mental images about the virus popped into my mind against my will. | 口 | 口 | 口 | 口 | 口 |
| 29. I had trouble concentrating because I kept thinking about the virus. | 口 | 口 | 口 | 口 | 口 |
| 30. Reminders of the virus caused me to have physical reactions, such as sweating or a pounding heart. | 口 | 口 | 口 | 口 | 口 |
| **The following items ask about checking behaviors. During the past seven days, how much have you checked the following because of concerns about COVID-19?** | Never | Rarely | Sometimes | Often | Almost always |
| 31. Social media posts concerning COVID-19. | 口 | 口 | 口 | 口 | 口 |
| 32. YouTube videos about COVID-19. | 口 | 口 | 口 | 口 | 口 |
| 33. Seeking reassurance from friends or family about COVID-19. | 口 | 口 | 口 | 口 | 口 |
| 34. Checking your own body for signs of infection (e.g., taking your temperature). | 口 | 口 | 口 | 口 | 口 |
| 35. Asking health professionals (e.g., doctors or pharmacists) for advice about COVID-19. | 口 | 口 | 口 | 口 | 口 |
| 36. Searched the Internet for treatments for COVID-19. | 口 | 口 | 口 | 口 | 口 |

*Appendix A.2. Chinese Version*

**TableA2** Items of the COVID Stress Scales (Chinese version)

| **指导语：以下情况可能是您在最近7天里各种经历过的担忧。请根据最近7天的情况，阅读每一道题目，选择符合您情况的选项。** | 没有 | 轻微 | 中等 | 较多 | 非常多 |
| --- | --- | --- | --- | --- | --- |
| 1. 我担心自己会感染新冠病毒。 | 口 | 口 | 口 | 口 | 口 |
| 1. 我担心基本卫生习惯(如洗手)不足以预防新冠病毒。 | 口 | 口 | 口 | 口 | 口 |
| 1. 我担心我们的医疗体系无法让我远离新冠病毒。 | 口 | 口 | 口 | 口 | 口 |
| 1. 我担心我不能让我的家人远离新冠病毒。 | 口 | 口 | 口 | 口 | 口 |
| 1. 我担心我们的医疗系统无法让我关心与爱的人远离新冠病毒。 | 口 | 口 | 口 | 口 | 口 |
| 1. 我担心保持社交距离不足以让预防新冠病毒。 | 口 | 口 | 口 | 口 | 口 |
| 1. 我担心商店的东西卖完。 | 口 | 口 | 口 | 口 | 口 |
| 1. 我担心药店里的感冒药或流感药卖完。 | 口 | 口 | 口 | 口 | 口 |
| 1. 我担心药店的处方药卖完。 | 口 | 口 | 口 | 口 | 口 |
| 1. 我担心商店的水卖完。 | 口 | 口 | 口 | 口 | 口 |
| 1. 我担心商店里的清洁和防病毒传染用品（例如：口罩）卖完。 | 口 | 口 | 口 | 口 | 口 |
| 1. 我担心商店关门。 | 口 | 口 | 口 | 口 | 口 |
| **指导语：请根据最近7天的情况，阅读每一道题目，选择符合您情况的选项。（疫情相关人员是指生活或途径中高风险地区者、参与一线疫情处置的医务人员、防疫工作者及其他相关人员等。**） | 没有 | 轻微 | 中等 | 较多 | 非常多 |
| 13. 我担心疫情相关人员会传播新冠病毒。 | 口 | 口 | 口 | 口 | 口 |
| 14. 如果我无意中遇到疫情相关人员，我会担心他们可能携带新冠病毒。 | 口 | 口 | 口 | 口 | 口 |
| 15. 我因疫情相关人员可能携带新冠病毒而担心与他们接触。 | 口 | 口 | 口 | 口 | 口 |
| 16. 如果疫情相关人员不注意卫生，所以我担心他们会传播新冠病毒。 | 口 | 口 | 口 | 口 | 口 |
| 17. 如果我去过疫情相关人员去过的餐厅吃饭，我担心自己感染新冠病毒。 | 口 | 口 | 口 | 口 | 口 |
| 18. 如果我和疫情相关人员同时乘坐电梯，我担心电梯上的人都感染了新冠病毒。 | 口 | 口 | 口 | 口 | 口 |
| 19. 我担心我周围的人会把新冠病毒传染给我。 | 口 | 口 | 口 | 口 | 口 |
| 20. 如果我在公共场所触摸到什么东西(如扶手、门把手)，我担心会感染新冠病毒。 | 口 | 口 | 口 | 口 | 口 |
| 21. 如果有人在我旁边咳嗽或者擤鼻涕，我担心会感染新冠病毒。 | 口 | 口 | 口 | 口 | 口 |
| 22. 我因使用现金或ATM机而担心会感染病毒。 | 口 | 口 | 口 | 口 | 口 |
| 23. 我担心用现金支付会拿到其他人接触过的零钱。 | 口 | 口 | 口 | 口 | 口 |
| 1. 我担心我的快递被新冠病毒污染了。 | 口 | 口 | 口 | 口 | 口 |
| **指导语：请根据最近7天的情况，阅读每一道题目，选择符合您情况的选项。** | 从不 | 很少 | 有时 | 经常 | 总是 |
| 25.我因担心新冠病毒而失眠。 | 口 | 口 | 口 | 口 | 口 |
| 26.我曾做过关于新冠病毒的噩梦。 | 口 | 口 | 口 | 口 | 口 |
| 27.我会在无意中想到新冠病毒。 | 口 | 口 | 口 | 口 | 口 |
| 28.关于新冠病毒的令人不安的画面无法控制地突然出现在我的脑海里。 | 口 | 口 | 口 | 口 | 口 |
| 29.我因一直在想新冠病毒而无法集中注意力。 | 口 | 口 | 口 | 口 | 口 |
| 30.有关新冠病毒的信息会让我身体产生反应，例如出汗或者心跳加快。 | 口 | 口 | 口 | 口 | 口 |
| **指导语：请根据最近7天的情况，阅读每一道题目，选择您因担心新冠疫情而查看了以下内容的频率。** | 从不 | 很少 | 有时 | 经常 | 总是 |
| 31.浏览社交媒体上关于新冠病毒的新闻。 | 口 | 口 | 口 | 口 | 口 |
| 32.观看有关新冠病毒的视频。 | 口 | 口 | 口 | 口 | 口 |
| 33.向朋友或家人反复确认疫情情况，以减少担心或疑虑。 | 口 | 口 | 口 | 口 | 口 |
| 34.检查身体是否有感染的症状(如量体温)。 | 口 | 口 | 口 | 口 | 口 |
| 35.寻求健康专家（例如：医生和药师）关于预防新冠病毒的建议。 | 口 | 口 | 口 | 口 | 口 |
| 36.在网上查询有关新冠肺炎的治疗方法。 | 口 | 口 | 口 | 口 | 口 |
